# Identification of candidate categories of the International Classification of Functioning Disability and Health (ICF) for a *Generic ICF Core Set *based on regression modelling

**DOI:** 10.1186/1471-2288-6-36

**Published:** 2006-07-27

**Authors:** Alarcos Cieza, Szilvia Geyh, Somnath Chatterji, Nenad Kostanjsek, Bedirhan T Üstün, Gerold Stucki

**Affiliations:** 1 ICF Research Branch of the WHO Collaborating Center for the Family of International Classifications at the German Institute of Medical Documentation and Information (DIMDI), IHRS, Ludwig-Maximilian University, Munich, Germany; 2 Swiss Paraplegic Research, Nottwil, Switzerland; 3 Classification, Assessment, Surveys and Terminology Team, World Health Organization, Geneva, Switzerland; 4 Department of Physical Medicine and Rehabilitation, Ludwig-Maximilian University, Munich, Germany

## Abstract

**Background:**

The International Classification of Functioning, Disability and Health (ICF) is the framework developed by WHO to describe functioning and disability at both the individual and population levels.

While condition-specific ICF Core Sets are useful, a Generic ICF Core Set is needed to describe and compare problems in functioning across health conditions.

**Methods:**

The aims of the multi-centre, cross-sectional study presented here were: a) to propose a method to select ICF categories when a large amount of ICF-based data have to be handled, and b) to identify candidate ICF categories for a Generic ICF Core Set by examining their explanatory power in relation to item one of the SF-36.

The data were collected from 1039 patients using the ICF checklist, the SF-36 and a Comorbidity Questionnaire.

ICF categories to be entered in an initial regression model were selected following systematic steps in accordance with the ICF structure. Based on an initial regression model, additional models were designed by systematically substituting the ICF categories included in it with ICF categories with which they were highly correlated.

**Results:**

Fourteen different regression models were performed. The variance the performed models account for ranged from 22.27% to 24.0%. The ICF category that explained the highest amount of variance in all the models was *sensation of pain*. In total, thirteen candidate ICF categories for a Generic ICF Core Set were proposed.

**Conclusion:**

The selection strategy based on the ICF structure and the examination of the best possible alternative models does not provide a final answer about which ICF categories must be considered, but leads to a selection of suitable candidates which needs further consideration and comparison with the results of other selection strategies in developing a Generic ICF Core Set.

## Background

Functioning is an important study outcome in relation to chronic health conditions. The number of studies addressing functioning as a study endpoint in patients with chronic conditions has steadily increased during the last decades.

In health outcome research, functioning is measured from different perspectives. In clinical research, functioning is assessed to describe the limitations and restrictions of patients before and after an intervention. In the field of quality-of-life research, functioning is assessed from the patients' perspective describing how patients feel about those limitations and restrictions. In economic evaluations, the personal value that the patients attribute to such limitations and restrictions is analyzed [1].

However, functioning is not only an outcome on end results of health services in relation to chronic health conditions. At the individual level, functioning represents the starting point from which to plan interventions [2]. At the institutional and social levels, functioning provides the basis for predicting need of care [3,4], length of treatment or hospitalization [5-10] and planning the distribution of resources.

The International Classification of Functioning, Disability and Health (ICF; [11]) is the framework developed by the World Health Organization (WHO) to describe functioning and disability at both the individual and population levels. The ICF represents the universal language of functioning to be used not only in outcome assessment, but also for planning health interventions and resources. Moreover, the ICF is intended to be used in multiple sectors that include, beside health, education, insurance, labour, health and disability policy and statistics [12].

**Figure 1:**
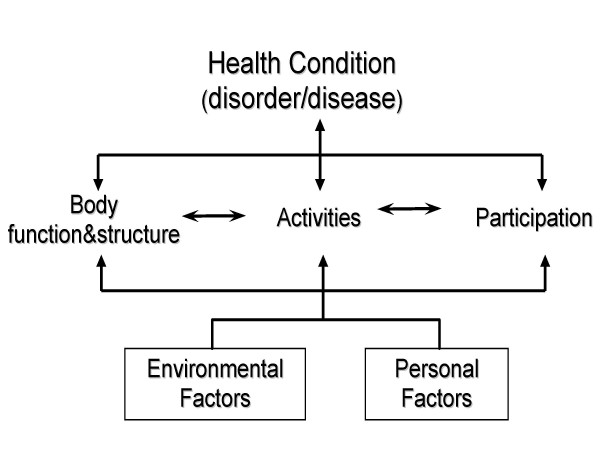
The current framework of Functioning, Disability and Health (ICF).

The development of the ICF was guided by a bio-psycho-social or integrative model of functioning and disability. Based on this model, functioning, with its components *Body Functions and Structures *and *Activities and Participation*, is seen in relation to the health condition under consideration, as well as personal and environmental factors (Fig. 1)[11]. Functioning denotes the positive aspects, and disability denotes the negative aspects of the interaction between an individual with a health condition and the contextual factors (environment and personal factors) of that individual. Thus, disability is an umbrella term for impairments, limitations in activities and restrictions in participation. This distinction can help when reading the medical literature. Disability is usually the preferred term. However, functioning is implicitly addressed when disability is studied and vice versa [13].

In 2001, 191 member states of the World Health Organization agreed to adopt the ICF as a basis for the scientific standardization of data on health and disability worldwide [14]. Since then, there is growing interest and even enthusiasm in the application of this classification in clinical practice and research [15,16]. However, it is now well understood that there are a number of challenges that need to be addressed to fully implement the ICF in everyday practice.

The ICF Core Sets represent one approach to operationalize the ICF for clinical practice and research. In the form of short, generally-agreed-on lists of ICF categories relevant for specific health conditions, the ICF Core Sets address a number of challenges that include the feasibility of the ICF and the links between functioning and specific health conditions, i.e. links between the ICF and the International Classification of Diseases (ICD-10) [16].

However, while the condition and setting- specific ICF Core Sets are useful when describing and classifying functioning for patients with specific health problems or in specific care settings, a *Generic ICF Core Set *is needed to describe and compare across health conditions. In other words a Generic ICF Core Set serves as a common currency and is a true operationalization of the ICF model as it applies the principle of etiologically neutrality. A *Generic ICF Core Set *will be developed in an iterative process involving a number of criteria and methodological approaches. One of these approaches will be the examination of the explanatory power of determined ICF categories in relation to external standards across the 12 chronic conditions for which condition-specific ICF Core Sets have already been developed.

Different approaches can be used to capture broad concepts, such as functioning and health. In the literature, it is traditionally distinguished between single questions and questionnaires capturing a number of domains [17].

The use of a single question or item representing a broad construct is an important consideration when deciding which external standard to include as independent variable in regression models. In contrast to a health profile or an index, which consist of several items, a single item provides a very broad [17] but straight-forward measure about the concept under consideration. The Short-Form 36 [SF-36; [18]] is the most widely-used health-status measure. Item one of this instrument is arguably one of the most widely-investigated single items referring to health in general [19]. Moreover, it has high face validity. Therefore, item one of the SF-36 questionnaire has been used for this investigation.

The general objective of the present study is to propose a number of candidate ICF categories for a *Generic ICF Core Set*.

The specific aims are: a) to propose a method to select ICF categories to be entered in a regression model when a large amount of ICF-based data have to be handled and b) to identify candidate ICF categories for a *Generic ICF Core Set *by examining the explanatory power of ICF categories in relation to item one of the SF-36 using regression modeling.

## Methods

### Study design

The study was a multi-centre, cross-sectional study.

The study protocol and informed-consent forms were approved by the Ethics Committee of the Ludwig-Maximilian University (LMU) of Munich, as well as the District Medical Council of Bavaria in Germany.

The methods of this study have previously been described in detail [20].

### Subjects

The study was performed with convenience samples of patients with at least one of the 12 chronic health conditions presented in Table 1 and undergoing inpatient or outpatient rehabilitation in 19 German clinics and rehabilitation centres.

**Table 1:** Index health conditions and number and percentage of patients with the health condition as main diagnosis

**Condition**	**n**	**%**
**Low back pain**	199	19
**Osteoporosis**	35	3
**Rheumatoid arthritis**	40	4
**Osteoarthritis**	62	6
**Coronary heart disease **	84	8
**COPD & Asthma**	92	9
**Diabetes Mellitus**	77	7
**Breast cancer**	119	11
**Obesity**	67	6
**Chronic widespread pain**	119	11
**Depressive disorder**	65	6
**Stroke**	116	11

Patients were included if the main diagnosis was one of the 12 index diagnoses (Table 1), they were at least 18 years old, had sufficient knowledge of the German language, understood the purpose and reason of the study, and had signed an informed consent.

Patients with secondary wound healing after surgery were excluded from the study.

### Measures

The ICF-based data were collected using the ICF checklist [21]. Like the ICF, the ICF checklist contains a list of 125 so-called ICF categories organized into three different components: (1) *Body Functions and Structures*, (2) *Activities and Participation*, and (3) *Environmental Factors*. *Personal Factors*, which constitute the fourth component of the classification and belongs to the part *Contextual Factors *as well, has not yet been classified.

The ICF categories are designated by the letters b (*Body Functions*), s (*Body Structures*), d (*Activities and Participation*), and e (*Environmental Factors*), followed by a numeric code starting with the chapter number (one digit), followed by the second level (two digits) and the third and fourth levels (one digit each). Within each component, the categories are arranged in a stem/branch/leaf scheme. Consequently, a higher-level category shares the attributes of the lower-level categories to which it belongs, i.e., the use of a higher-level (more detailed level) category automatically implies that the lower-level category is applicable.

The ICF checklist contains first- and second-level ICF categories. With respect to all categories on the second level of the ICF, the ICF Checklist includes 29 (25%) categories from the component *Body Functions*, 16 (29%) from *Body Structures*, 48 (41%) from *Activities and Participation*, and 32 (43%) from *Environmental Factors*.

To evaluate the extent of the patient's problem in each of the ICF categories, so-called generic qualifier scale was used. The qualifier scale of the components *Body Functions*, *Body Structures *and *Activities and Participation *has five response categories, each ranging from 0 to 4: no/mild/moderate/severe/complete impairment or difficulty. The qualifier scale of the component Environmental Factors has nine response categories ranging from -4 to +4. A specific environmental factor can be a barrier (-1 to -4), a facilitator (1 to 4), or can have no influence (0) on the patient's life. If the factor has an influence, the extent of the influence (either positive or negative) can be coded with mild/moderate/severe/complete. In addition, there are the response options "8 – not specified" and "9 – not applicable" [11].

In this study, broad ranges of percentages as they are provided by WHO [11] were used as a reference system to quantify the problems of the patients in each of the different ICF categories and the extent to which a determined environmental factor was a barrier or a facilitator.

0 – NO problem (none, absent, negligible,...) 0–4%

1 – MILD problem (slight, low,...) 5–24%

2 – MODERATE problem (medium, fair...) 25–49%

3 – SEVERE problem (high, extreme,...) 50–95%

4 – COMPLETE problem (total,...) 96–100%

The **SF-36 **[18] derives from a large battery of questions administered in the Medical Outcomes Study. The SF-36 includes eight multi-item scales containing 2–10 items each and a single item to assess health transition between two different time points of assessment. Two summary scales can also be obtained – the Physical Component Summary Score (**PCS**) and the Mental Component Summary Score (**MCS**). The first item of the questionnaire addresses health in general and reads: "In general, would you say your health is (excellent/very good/good/fair/poor)?".

Empirical work has consistently shown that this item requires recalibration, since the intervals between adjacent response categories are unequal. Therefore, the item scale values are transformed as follows: excellent = 5.0, very good = 4.4, good = 3.4, fair = 2.0 and poor = 1.0 [19]. The transformed data were used for the data analyses in this study.

The **SCQ **is an instrument to assess comorbidity for clinical and health-services research. The patients are first asked whether they have problems with each of the following health conditions or not: (1) heart disease, (2) high blood pressure, (3) lung disease, (4) cancer, (5) depression, (6) arthritis and (7) back pain. If the answer is yes, the patients are additionally asked whether they are receiving treatment for it or not. To assess the burden of disease on the patient, s/he is asked whether the problem limits their activities or not. The subjects can also add three additional health conditions. The number of diseases as measured by the SCQ was used as a control variable in the regression models of this study.

### Data collection

The recruitment of the patients and ICF-based data collection were performed by physicians and other health professionals trained by researchers of the ICF Research Branch WHO FIC CC (ICF Research Branch, World Health Organization, Family of International Classifications' Collaborating Center) in a structured one-day workshop.

During the training, all participants were familiarized with the ICF framework and classification and provided with instructions for data collection. The ranges of percentages used as references to quantify the problems of the patients in each of the different ICF categories were also introduced and explained. An exemplary case was provided to practice the data collection.

The self-administered forms of the SF-36 and the Comorbidity Questionnaire (SCQ, [22]) were filled in by the patients on their own. Health professionals were available to answer questions.

### Analysis

Descriptive statistics were used to define the study population and describe the health status of the patients based on the eight subscales of the SF-36.

As the ICF qualifiers "8 – not specified" and "9 – not applicable" cannot be integrated in the ordinal scale of the ICF qualifiers 0 to 4 and -4 to 4, respectively, they were deleted from the database and considered missing values. Missing values were then replaced by the Expectation-Maximization algorithm or EM algorithm, a maximum likelihood method [23]. This method does not underestimate variance, as is common in replacement by mean. However, this more conservative method was used to validate the results from the analyses with the EM-algorithm. The control analyses led to the selection of identical variables

### Selection of ICF categories

The selection of the ICF categories to be entered in an initial regression model occurred in three steps. **First**, according to the descriptive statistics on the ICF categories, only those ICF categories representing a problem for at least 10% of the patients were considered for further analyses. In Environmental Factors, the ICF category had to represent a barrier or facilitator for at least 10% of the patients.

**Second**, there had to be a substantial relationship to general health as measured by item 1 of the SF-36. The relationship was analysed by the Spearman correlation coefficient. The correlation had to show a probability value lower than .01.

**Third**, the requirement of independence of the variables was analyzed using again the Spearman correlation coefficient. The inter-correlations of the ICF categories that belong to the same ICF chapters were analysed before considering the inter-correlations of ICF categories that belong to different chapters.

**Figure 2:**
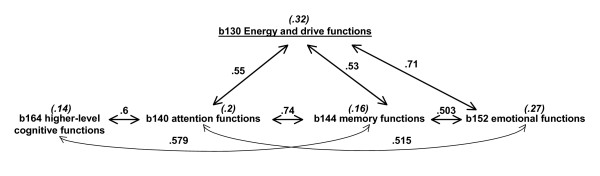
Inter-correlated ICF categories in chapter 1 *mental functions*. Values next to the arrows represent the inter-correlations of the ICF categories. Values between brackets and cursive represent the correlation of the corresponding ICF category with item 1 of the SF-36.

For inter-correlations between/among two or more ICF categories of the same chapter above 0.5, the ICF category with the highest correlation with item 1 of the SF-36 was further considered. For example, the inter-correlations among the ICF categories that belong to chapter *mental functions *are presented in Fig. 2. From all these inter-correlated ICF categories, the ICF category *b130 energy and drive functions *was further considered, since it is the category with the highest correlation with item 1 of the SF-36.

**Figure 3:**
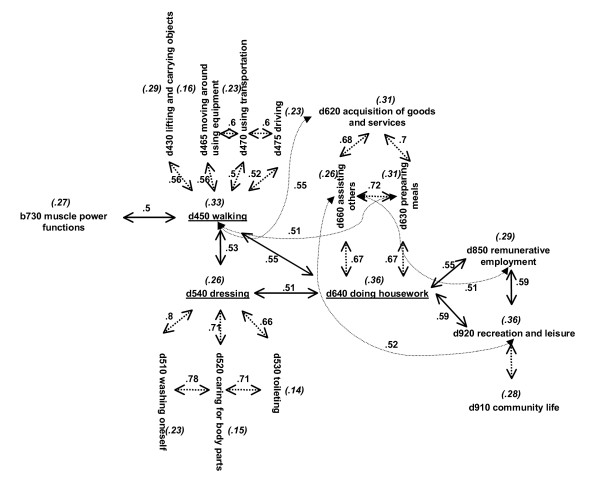
Inter-correlated ICF categories in the component *body functions *and *activities and participation*. The discontinued arrows refer to inter-correlations among/between the ICF categories that belong to the same ICF chapter. The continued arrows refer to inter-correlations of ICF categories from different chapters. Values next to the arrows represent the inter-correlations of the ICF categories. Values between brackets and cursive represent the correlation of the corresponding ICF category with item 1 of the SF-36.

For inter-correlations between/among two or more ICF categories of different chapters above 0.5, the ICF category with the highest correlation with item 1 of the SF-36 was further considered. For example, the inter-correlations among the ICF categories within and among the chapters 4, 5 and 6 are shown in Fig. 3. Since *d450 walking*, *d540 dressing *and *d640 doing housework *are the ICF categories with the highest correlation with item 1 of the SF-36 in their respective chapters, they were further considered. However, since these three categories were inter-correlated, only the ICF category *d640 doing housework *was selected to be included in the regression analyses, since it is the category with the highest correlation with item 1 of the SF-36.

### Test of the homoscedasticity assumption

To verify whether the data met the regression assumption of homoscedasticity, the residuals were plotted against the predicted values for each of the regression analyses performed (see below). Obvious departures from the homoscedasticity assumption were consistently detected and consequently a White test [24] conducted. The variance of the residuals proved always not to be homoscedastic based on the White test.

Consequently, all regression analyses presented in the paper were performed using a Heteroscedasticity-Consistent Standard Error (HCSE) estimator of ordinary least square regression [25-27]. With this method, the regression model is estimated using ordinary least squares, but the standard errors are estimated without imposing a constraint (either assumed or modelled) on the structure of the errors. We used the most frequently used and most recommended HCSE estimator known as HC3 [25,28].

### Development of an initial regression model

An initial regression model explaining item 1 of the SF-36 was developed again in 3 steps: In **step 1**, four different regression analyses were performed, each of which included the selected ICF categories belonging to an ICF component (*Body Functions*, *Body Structures*, *Activities/Participation*, and *Environmental Factors*). A multiple-linear model using the HC3 estimator with stepwise selection with p < .05 for inclusion and exclusion of a variable was used for these four regression analyses.

In **step 2**, the variables selected in the four regression analyses in the previous step were integrated into one multiple-linear regression model explaining general health as measured by item 1 of the SF-36. The variables indicating the presence or absence of the 12 different index health conditions that were correlated to item 1 of the SF-36 were also included in this model. Again, stepwise selection using the HC3 estimator with p < .05 for inclusion and exclusion of a variable was used.

In **step 3**, three control variables were included in the model, namely, gender, age, and number of concomitant diseases.

### Development of additional regression models

Additional regression models explaining item 1 of the SF-36 were designed based on the initial regression model by systematically substituting the ICF categories included in it with ICF categories with which they were highly correlated. Both, Inter-correlations within the same chapters and inter-correlations of ICF categories belonging to different chapters were considered. For each of the additional regression models, the same 3 steps that were performed for the initial regression model were followed.

## Results

1039 patients from 19 German rehabilitation centers were included in the study. 58.6% of the patients were female. Patients were between 18 and 84 years (mean age: 53 years). Two thirds of the patients (66.3%) were married, and 62.4% were paid/non-paid employment (self-employed, students, do housework). 25.3% were retired, and 7.6% were unemployed.

Information on diagnoses and health status as measured by the SF-36 are shown in Tables 1 and 2, respectively.

**Table 2:** Descriptive statistics SF-36 (scales and summary measures, N = 1040)

**SF-36**	**N**	**Min**	**Max**	**Mean**	**Std**
**Scales**					
**Physical Functioning**	1021	0.0	100.0	59.9	27.8
**Role Physical**	990	0.0	100.0	36.0	40.4
**Bodily Pain**	1019	0.0	100.0	47.2	29.5
**General Health**	999	0.0	100.0	48.4	19.3
**Vitality**	1005	0.0	100.0	41.9	20.9
**Social Functioning**	1023	0.0	100.0	66.0	28.5
**Role Emotional**	971	0.0	100.0	62.7	44.5
**Mental Health**	995	0.0	100.0	59.1	21.8

**Summary Measures**					
**Physical Health Index Score**	933	7.2	70.0	37.2	11.0
**Mental Health Index Score**	933	9.0	73.8	44.5	13.1

Patients with 12 different health conditions were included in the study. The largest patient group suffered from low back pain (19.2% of the patients). Osteoporosis is the least frequent diagnosis (3.4%).

About one third of the subjects had only one disease. The number of diseases suffered by the patients ranged between 1 and 13 diseases. 76.4 % of the patients reported 1 to 3 different diseases. The average number of diseases was 2.2.

In the SF-36, the lowest health status was reported for the scale *Role Physical *and *Vitality*. The highest health status was in the scales *Role Emotional *and *Social Functioning*.

In the summary scales, patients reported stronger limitations in the PHI score than in the MHI score.

28 ICF categories were considered for the initial regression model after having analyzed which categories represented a problem for at least 10% of the patients, their correlation to the item 1 of the SF-36 and the requirement of independence.

In addition to the initial model, 13 different regression models were performed by substituting the ICF categories included in the initial model with ICF categories with which they were highly correlated. Table 3 and 4 show the results of the 14 different regression models applied. The first line of Tables 3 and 4 shows which ICF categories of the initial model were substituted by which other ICF categories.

Table 3 displays the R^2 ^or the amount of variance accounted for by the single independent variables and the total variance in the different models. Table 4 displays the parameter estimates, the estimated heteroscedasticity-consistent standard errors and the corresponding p-values for each of the dependent variables of the different 14 models.

**Table 3:** R^2 ^or the amount of variance the single independent variables and the total variance the corresponding 14 models account for and number of ICF categories resulting from each of the different steps of the selection of ICF categories.

**Item 1 of the SF-36**	**Initial Model**	**Model II b140 instead b130 & b164**	**Model III b152 instead b130**	**Model IV b144 instead b130 & b164**	**Model V d450 & d920 instead d640**	**Model VI d475 & d920 instead d640**	**Model VII d470 & d920 instead d640**	**Model VIII d450 & d850 instead d640**	**Model IX d475 & d850 instead d640**	**Model X d470 & d850 instead d450**	**Model XI d540 & d920 instead d640**	**Model XII d620 instead d640**	**Model XIII d630 instead d640**	**Model XIV d660 instead d640**
Age	0.0345	0.0346	0.0346	0.0346	0.0351	0.035	0.0353	0.0352	0.0302	0.0351	0.0351	0.0346	0.0344	0.0346
Gender														
Comorbidities (N)														

*b130 Energy and drive functions*	0.0498				0.0449	0.0445	0.046	0.0479	0.0437	0.0456	0.0449	0.0166	0.0497	0.0449
b152 Emotional Functions			0.0125											
b235 Vestibular functions											0.0065			
b280 Sensation of pain	0.1073	0.1142	0.1157	0.1119	0.1177	0.1172	0.1169	0.1117	0.1096	0.1113	0.1135	0.1082	0.1086	0.1143
b730 Muscle power functions											0.0043			
d450 Walking					0.019			0.0184						
D620 Acquisition of goods and services												0.0470		
*d640 Doing housework*	0.0191	0.0507	0.0512	0.0561										
d660 Assisting others														0.0065
d850 Remunerative employment									0.0110	0.0104				
d920 Recreation & Leisure						0.0143								
e450 Individual attitudes of health professionals													0.0060	
e580 Health services, systems and policies														0.0051
Breast cancer	0.0123	0.0128	0.0133	0.0131	0.0078	0.01	0.0134	0.0081	0.0141	0.0140	0.0139	0.0134	0.0138	0.0139
Osteoporosis	0.0095	0.0117	0.0114	0.0117	0.0075	0.0089	0.0097	0.0075	0.0141	0.0085	0.0094	0.0109	0.0116	0.0121
Chronic widespread pain				0.0039										
Depressive disorder		0.0088		0.0086										
Diabetes Mellitus							0.0051						0.0058	

**Total R2**	**0.2325**	**0.2328**	**0.2387**	**0.2399**	**0.232**	**0.2299**	**0.2264**	**0.2288**	**0.2227**	**0.2249**	**0.2276**	**0.2307**	**0.2299**	**0.2314**

**Table 4:** Parameter estimates (PE), estimated heteroscedasticity-consistent standard errors (SE) and corresponding p-values (p) for each of the dependent variables of the different 14 models.

**Item 1 of the SF-36**		**Model I**	**Model II b140 instead b130 & b164**	**Model III b152 instead b130**	**Model IV b144 instead b130 & b164**	**Model V d450 & d920 instead d640**	**Model VI d475 & d920 instead d640**	**Model VII d470 & d920 instead d640**	**Model VIII d450 & d850 instead d640**	**Model IX d475 & d850 instead d640**	**Model X d470 & d850 instead d450**	**Model XI d540 & d920 instead d640**	**Model XII d620 instead d640**	**Model XIII d630 instead d640**	**Model XIV d660 instead d640**
Intercept	PE	2.97	3.03	2.96	3.04	2.93	2.95	2.91	2.96	2.93	2.94	2.94	2.91	2.90	2.93
	SE	0.1687	367.79	372.05	372.39	346.81	352.23	331.20	349.90	346.28	348.48	350.57	354.74	338.34	358.11
	p	<.000	<.0001	<.0001	<.0001	<.0001	<.0001	<.0001	<.0001	<.0001	<.0001	<.0001	<.0001	<.0001	<.0001
Age	PE	0.002	0.002	0.002	0.001	0.002	0.001	0.000	0.002	0.001	0.001	0.001	0.003	0.001	0.001
	SE	0.003	0.003	0.003	0.003	0.003	0.003	0.003	0.003	0.003	0.003	0.003	0.003	0.003	0.003
	p	0.477	0.409	0.548	0.664	0.456	0.663	0.999	0.479	0.758	0.784	0.689	0.361	0.628	0.621
Gender	PE	-0.018	0.001	0.007	-0.031	0.023	0.041	0.037	0.024	0.050	0.052	0.022	-0.001	0.031	0.043
	SE	0.065	0.063	0.063	0.065	0.064	0.064	0.064	0.065	0.065	0.064	0.064	0.064	0.063	0.063
	p	0.776	0.992	0.908	0.631	0.719	0.524	0.566	0.710	0.440	0.420	0.731	0.984	0.625	0.495
Comorbidities (N)	PE	-0.086	-0.095	-0.091	-0.089	-0.094	-0.096	-0.094	-0.093	-0.095	-0.092	-0.091	-0.086	-0.092	-0.088
	SE	0.023	0.023	0.022	0.023	0.024	0.024	0.024	0.024	0.024	0.024	0.023	0.023	0.023	0.022
	p	0.000	0.000	0.000	0.000	0.000	0.000	0.000	0.000	0.000	0.000	0.000	0.000	0.000	0.000
*b130 Energy and drive functions*	PE	-0.140				-0.161	-0.140	-0.190	-0.168	-0.146	-0.146	-0.188	-0.122	-0.176	-0.144
	SE	0.034				0.033	0.034	0.032	0.033	0.035	0.035	0.033	0.034	0.032	0.035
	p	0.000				0.000	0.000	0.000	0.000	0.000	0.000	0.000	0.000	0.000	0.000
**b152 Emotional Functions**	PE			-0.125											
	SE			0.038											
	p			0.001											
b235 Vestibular functions	PE											0.142			
	SE											0.063			
	p											0.023			
b280 Sensation of pain	PE	-0.208	-0.227	-0.222	-0.203	-0.230	-0.234	-0.246	-0.226	-0.220	-0.231	-0.219	-0.222	-0.219	-0.226
	SE	0.027	0.027	0.026	0.028	0.027	0.027	0.026	0.027	0.027	0.026	0.027	0.026	0.025	0.026
	p	0.000	0.000	0.000	0.000	0.000	0.000	0.000	0.000	0.000	0.000	0.000	0.000	0.000	0.000
b730 Muscle power functions	PE											-0.096			
	SE											0.034			
	p											0.005			
**d450 Walking**	PE					-0.110			-0.108						
	SE					0.037			0.037						
	p					0.003			0.004						
**d620 Acquisition of goods and services**	PE												-0.134		
	SE												0.032		
	p												0.000		
*d640 Doing housework*	PE	-0.120	-0.174	-0.135	-0.178										
	SE	0.032	0.029	0.032	0.030										
	p	0.000	0.000	0.000	0.000										
**d660 Assisting others**	PE														-0.073
	SE														0.032
	p														0.022
d850 Remunerative employment	PE									-0.065	-0.062				
	SE									0.020	0.022				
	p									0.001	0.004				
d920 Recreation & Leisure	PE						-0.090								
	SE						0.031								
	p						0.004								
e450 Individual attitudes of health professionals	PE													-0.066	
	SE													0.023	
	p													0.004	
e580 Health services, systems and policies	PE														-0.053
	SE														0.023
	p														0.020
Breast cancer	PE	0.381	0.356	0.403	0.323	0.317	0.349	0.394	0.322	0.423	0.414	0.392	0.398	0.454	0.384
	SE	0.087	0.089	0.088	0.091	0.093	0.090	0.089	0.093	0.089	0.088	0.088	0.088	0.089	0.089
	p	0.000	0.000	0.000	0.000	0.001	0.000	0.000	0.001	0.000	0.000	0.000	0.000	0.000	0.000
Osteoporosis	PE	0.526	0.563	0.576	0.521	0.479	0.519	0.543	0.479	0.528	0.508	0.591	0.561	0.570	0.522
	SE	0.142	0.145	0.139	0.146	0.146	0.145	0.145	0.146	0.146	0.144	0.148	0.145	0.144	0.140
	p	0.000	0.000	0.000	0.000	0.001	0.000	0.000	0.001	0.000	0.001	0.000	0.000	0.000	0.000
Chronic widespread pain	PE				-0.231										
	SE				0.097										
	p				0.017										
Depressive disorder	PE		-0.375		-0.399										
	SE		0.127		0.130										
	p		0.003		0.002										
Diabetes Mellitus	PE													0.298	
	SE													0.126	
	p													0.019	

The initial model includes eight independent variables, from which three are the fixed control variables (age, gender and number of diseases), and the presence vs. absence of the diagnosis breast cancer and osteoporosis as the main diagnosis. The ICF categories included in this first model are *b130 energy and drive functions*, *b280 sensation of pain*, *d640 doing housework*.

*B280 sensation of pain *did not correlated above 0.5 with any other variable. Therefore, this category was not substituted for any other category in the regression model conducted after the initial model.

Models II to IV were based on the variables that correlated above 0.5 with *b130 energy and drive functions *(see Fig. 2). *B130 energy and drive functions *was substituted for *b140 attention functions *in model II, for *b152 emotional functions *in model III and for *b144 memory functions *in model IV, respectively. In models II and IV, the ICF category *b164 higher-level cognitive functions *was also excluded from the analyses since this ICF category correlated above 0.5 with *b140 attention functions *and *b144 memory functions*.

The ICF categories *b140 attention functions *and *b144 memory functions *did not significantly explain any variance in item 1 of the SF-36 and did not, therefore, remain in their respective models. However, other variables, such as the presence vs. absence of depressive disorder and chronic widespread pain, significantly explained 0.9 and 0.4 of the variance of the item 1 of the SF-36 and, therefore, remained in the respective models.

The ICF category *b152 emotional functions *significantly explained 1.2% of the variance in the item 1 of the SF-36 and remained in the model III. Model I and III included similar ICF categories. It is, however, interesting to note that *d640 doing housework *explained more variance in model III, in which the ICF category *b152 emotional functions *was included instead of *b130 energy and drive functions*.

Model V substituted the ICF category *d640 doing housework *for *d450 walking*, which correlated above 0.5 (see Fig. 3). Since *d450 walking *did not correlate to *d920 recreation and leisure *(as *d640 doing housework *does), *d920 recreation and leisure *was also included in the model. Otherwise, the ICF category *b730 muscle functions *was not included in the model because this ICF category correlated above 0.5 with *d450 walking*.

*D450 waking *remained in model V, significantly explaining 2% of the variance of item 1 of the SF-36.

Models VI to X are based on model V and in the inter-correlations presented in Fig. 3. In models VI and IX, *d450 walking *was substituted for *d475 driving *and in models VII and X, for *d470 using transportation*, respectively (see Fig. 3). In model VI and VII, the ICF category *d920 recreation and leisure *was included. In models VIII, IX and X the ICF category *d850 remunerative employment *was included.

The ICF categories *d475 driving *and *d470 using transportation *did not remain in the corresponding models VI, VII, IX and X. The ICF category *d920 *remained in model VI, significantly explaining 1.4 of the variance of the dependent variable. D*850 remunerative employment *remained in models IX and X explaining significantly 1.1 and 1% of the variance of the dependent variable. Model VIII was almost identical to model V.

Model XI substituted *d640 doing housework *for *d540 dressing *and *920 recreation and leisure*. Model XII substituted *d640 doing housework *for *d620 acquisition of goods and services*. Models XIII substituted *d640 doing housework *for *d630 preparing meals*. Model XIV substituted *d640 doing housework *for *d660 assisting others*. The ICF categories *d620 acquisition of goods and services *and *d660 assisting others *remained in their respective models, significantly explaining 4.7 and 0.6 % of the variance in the item 1 of the SF-36, respectively. It is important to mention that the R^2 ^of *b130 energy and drive functions *decreased to 0.017 when *d620 acquisition of goods and services *was included in the model.

The variance of the item 1 in the SF-36, the performed models account for, ranged from 22.27% in model IX to 24.0% in model IV. The control variables accounted for 3% to 3.5% of the variance in the dependent variable. However, only the variable *number of diseases *revealed a significant result (p < .0001) in all the models. Age and gender were not significant in any of the models.

The ICF category that explained the highest amount of variance in all the models (from 10.7% in model I to 11.8% in model V) was *b280 sensation of pain*.

The variables presence vs. absence of breast cancer and osteoporosis, respectively, were consistently present in all the models, explaining around 1% of the variance in the dependent variable. In all the models these two variables had positive parameter estimates, i.e., patients in these diagnosis groups with breast cancer and osteoporosis reported better general health than patients with other diseases as main diagnoses.

In all the models, the ICF categories and the variable number of diseases had consistently negative parameter estimates, i.e., high difficulties in these ICF categories (or high number of diseases, respectively) were accompanied by low general health as measured by item 1 in the SF-36.

## Discussion

In this study, a number of candidate ICF categories for a *Generic ICF Core Set *were proposed based on the examination of their explanatory power in relation to general health as defined by question one of the SF-36 using regression modeling. These categories were: *b130 energy and drive functions, b152 emotional functions, b230 vestibular functions, b280 sensation of pain, b730 muscle power functions, d450 walking, d620 acquisition of goods and services, d640 doing housework, and d660 assisting others, d850 remunerative employment, d920 recreation and leisure, e450 individual attitudes of health professionals, and e580 health services, systems and policies *(see also table 5).

**Table 5:** Overlap between 13 candidate ICF categories for Generic ICF Core Set and the WHO DAS II 12 item version

**ICF Component**	**Candidate ICF categories for Generic ICF Core Set**	**WHO DAS II 12 item version**
**Body Functions**	b130 energy and drive functions	BFs are currently not included. However, BF module is under consideration for development.
	b152 emotional functions	
	b230 vestibular functions	
	b280 sensation of pain	
	b730 muscle power functions	

	d450 walking	D1.1 Concentrating on doing something for ten minutes?
		D1.4 Learning a new task, for example, learning how to get to a new place?
		D2.1 Standing for long periods such as 30 minutes?
		D2.5 Walking a long distance such as a kilometre (or equivalent)?
		D3.1 Washing your whole body?
		D3.2 Getting dressed?
		D4.1 Dealing with people you do not know?
		D4.2 Maintaining a friendship?
**Activity & Participation**	d620 acquisition of goods and services	
	d640 doing housework	D5.1 Taking care of your household responsibilities?
	d660 assisting others	
	d850 remunerative employment	D5.5 Your day to day work/school?
	d920 recreation and leisure	D6.1 How much of a problem did you have in joining in community activities (for example, festivities, religious or other activities) in the same way as anyone else can?
		D6.5 How much have you been emotionally affected by your health condition

**Environmental Factors**	E450 individual attitudes of health professionals E580 health services, systems and policies	One generic EF question is currently included in d450 walking WHO DAS 36 item version. However, a EF Module is under consideration for development

BF = Body Function.

EF = Environmental Factor

With the exception of *d235 vestibular functions*, *d620 acquisition of goods and services*, *d660 assisting others*, and the two categories in the component *environmental factors*, the suggested ICF categories were addressed in at least 4 of the 6 most widely used generic health-status measures [29].

With the exception of *d235 vestibular functions*, which was listed in none, and *d660 assisting others*, which was listed in only 6 of the 12 condition-specific Comprehensive ICF Core Sets that have been developed so far, all the other candidate ICF categories were listed in at least 10 condition-specific Comprehensive ICF Core Sets [15]. Since the Comprehensive ICF Core Sets were developed to guide multidisciplinary assessments [30] in clinical settings like rehabilitation, they are relatively large. Thus, Brief ICF Core Sets representing a selection of ICF categories of the respective Comprehensive ICF Core Sets and addressing only those aspects of functioning that are essential and can be recorded in clinical studies were also developed. Within this context it is important to mention that *b130 energy and drive functions, b152 emotional functions*, *b280 sensation of pain*, *b730 muscle power functions, d450 walking*, *d640 doing housework*, and *e580 health services, systems and policies *are included in at least 6 of the 12 Brief ICF Core Sets developed up to now.

In addition, the 13 candidate ICF categories have high face validity, if one considers that a *Generic ICF Core Set *will have to include ICF categories which represent problems affecting most patients irrespective of their specific health conditions. As all others ICF Core Sets developed so far [30], a *Generic ICF Core Set *should include as few categories as possible to be practical, but as many as necessary to be sufficiently comprehensive to describe the patients' typical spectrum of problems in functioning across conditions.

The 13 candidate ICF categories already show a considerable overlap with the 12-Item version of the World Health Organization Disability Assessment Schedule II (WHODASII) (see Table 5). [31,32], which is an instrument with from the same conceptual basis as the ICF. Therefore, it is expected that the results from this and future work on the generic ICF Core Set will contribute to the further development of the WHODAS II, as well as to the development of ICF-based assessment instruments.

Instead of regression modelling, other methods, such as simply rank the strength of the association after using parametric or non-parametric correlation statistics (Sperman or Person Coefficients) could be considered when selecting ICF categories in relation to a broad concept, like health. However, regression modelling enables a better understanding of the relative contribution of individual ICF categories in relation to others [33].

In addition, a transparent selection process which acknowledges that within a set of highly-related variables not only the finally-selected variable, but also other variables would have similarly contributed to a model, is necessary to thoroughly study the relationship among possible candidate ICF categories. We developed a selection strategy based on the ICF structure and examined a number of alternative models, considering best possible alternatives from our selection strategy. This process does not lead to a final answer about which ICF category to consider or not, but at least leads to a selection of candidates which needs further consideration and comparison with the results of other selection strategies in developing a *Generic ICF Core Set*.

The importance of not relying on a purely statistical selection was demonstrated by the example *b152 emotional functions*. If one had simply followed the statistical modelling, *b152 emotional functions *would not have been selected in a final model and would not have been considered as a candidate ICF category for the generic ICF Core Set. This would be counterintuitive for most health professionals, since emotional functions in the personal experience of health professionals and patients are relevant domains that always must be taken into account to describe functioning, disability and health. According to Cieza and Stucki (2004; 29) all 6 most widely used generic health-status measures address emotional functions.

As can be seen in Fig. 2, *b130 energy and drive functions *are highly related to *b152 emotional functions *and vice versa. In a model including *b152 emotional functions *instead of *b130 energy and drive functions*, the total variance explained is similar, and all the variables entered are the same. A similar example can be seen with *d450 walking *which, when tracked back in the selection process, was highly associated with *d640 doing housework*. A model including *d450 walking *(model V) instead of *d640 doing housework *(model I) provides virtually a similar answer with regard to the variance explained and the other variables included. D620 *acquisition of goods and services *also appears to be a suitable substitute for *d640 doing housework*. However, category *b130 energy and drive functions *lose some importance when d620 is in the model.

It is also interesting to note that *d920 recreation and leisure *significantly explains some of the variance in general health when neither *d640 doing housework *nor *d450 walking *is in the same model. Taking into account that all the different models explain a similar amount of variance in the end, this indicates that all these variables explain the same variance in general health as measured by item one in the SF-36. The same can be said of d850 remunerative employment. This variable remains in the model when neither *d640 doing housework *nor *d450 walking *is in it.

Pain is the most relevant independent variable in all the models. Its importance remains the same, regardless of which variables are additionally included in the model. This result is not surprising, since pain is a leading symptom and one of the key outcomes in many different chronic conditions [34-43].

*Body structures *do not play a significant role in any of the models when explaining general health. This is probably because the organ systems involved in the health conditions affecting the patients in this study are very diverse. The fact that environmental factors are present in only two models is, however, remarkable. Environmental factors are defined as the physical, social and attitudinal environment in which people live and conduct their lives [11]. They represent factors that have an important influence on patients' health, and one would have expected more of them in the different models. This result should, therefore, be studied in similar studies and reflects the importance of an iterative procedure and the involvement of different criteria and methodological approaches for the development of a final *Generic ICF Core Set*.

Concerning the control variables, only the number of comorbidities has a significant influence on item 1 of the SF-36 in these analyses. This result is consistent through all the models. Individuals with fewer diseases feel healthier.

This study also presents several limitations that require special annotation.

It has to be recognized that the significance threshold of <0.01 that was needed to deal with the vast number of variables and that was set in the second step of the selection process is low. This might lead to the exclusion of potentially valuable ICF categories from the outset. This limitation is also in line with other limitations of the study, i.e., that our data were based in the ICF categories included in the ICF Checklist. As we now know from the development of the Comprehensive ICF Core Sets, a number of other categories not included in the ICF checklist, such as *b455 Exercise tolerance functions*, are relevant for 10 of the 12 health conditions considered. Regarding the Brief ICF Core Sets, the categories *b455 Exercise tolerance functions, d240 Handling stress and other psychological demands *and *d230 Carrying out daily routine *are included in seven, seven and five of the Brief ICF Core Sets, respectively, but are not included in the ICF checklist. Whether such categories will be included in the definitive Generic ICF Core Set will be investigated in the future.

A major limitation of our study is the relatively low variance explained by the ICF categories. There are a number of explanations to be considered which are relevant in the process of developing a *Generic ICF Core Set*. First of all, our selection was based only on the categories included in the ICF Checklist. Second, personal factors are still lacking within the scope of the ICF. However, personal factors refer to variables, such as fitness, lifestyle, social background and coping styles that definitely influence and determine general health. It has to be taken into account that, in this analysis, only the variables sex, age and number of concomitant diseases were included. Future studies should also include further personal factors as relevant independent variables. Third, no interaction terms were included in the models since the purpose of the study was simply to propose a method to select ICF categories and to identify candidate ICF categories for a *Generic ICF Core Set*. However, it is possible that interaction terms including, for example, *b130 energy and drive functions *and *b280 sensation of pain*, contribute to general health as measured by the item 1 of the SF-36. The interaction terms should be included in future studies. Four, and probably most important, in our patient sample there was not much variance in most of the categories, i.e., in many categories most people had no limitations. According to the guidelines that establish what kind of patients have access to rehabilitation after discharge from an acute hospital in Germany, all the patients have to be able to eat and wash themselves without external support, as well as be able to move independently on the ward. Therefore, one can assume that the limitations in functioning suffered by the patients included in this study do not represent the whole spectrum of severity of limitation in functioning of patients suffering from chronic conditions.

This limitation points out an additional limitation of the study – the generalization of the results. Only patients treated in rehabilitation centers in Germany were included in this study. Thus, patients treated in acute hospitals, day clinics and outpatients and inpatients in countries other than Germany are not represented. This again emphasizes the importance of performing similar analyses with patients in different countries who are being treated in different settings. This will be possible with the data collected in the international validation study of the ICF Core Sets involving over 50 countries and 270 study centres that is being performing until the end of 2006.

It has also to be taken into account that no further operationalisation of the qualifier scale besides the broad ranges of percentages provided by WHO were used in this study. The ICF checklist in its current form contains a more detail description of the qualifiers. For example, in the component *body functions 1 *(*mild impairment) *is defined as "*a problem that is present less than 25% of the time, with an intensity a person can tolerate and which happens rarely over the last 30 days"*. The descriptions of the ICF qualifiers were not available at the time when the data collection of the study presented here was planed and carried out. Future studies should include the actual descriptions of the qualifier scale, since they may improve the reliability and validity of the data. Within this context it is important to mention that reliability studies are being currently performed at the ICF Research Branch at the Ludwig-Maximilian University to study the psychometric properties of the qualifier scale.

The SF-36 summary scores of the persons participating in this study averaged approximately 13 and 7 points less than the German normal population on physical and mental health, respectively. There are no substantial differences when compared to the German reference population with chronic conditions [44].

The fact that patients reported greater limitations in the PHI score than in the MHI score of the SF-36 is not surprising, as only 65 of the 1039 patients in our sample had a health condition that is traditionally considered as a mental-health related condition, namely depression. It is also important to mention that the German reference population with chronic conditions does not include mental-health related conditions.

In our study, we controlled for the presence/absence of each single health condition, including depression, in all performed regression models. However, the disproportionate higher number of patients with physical-health conditions makes it difficult to generalize our results to patients with mental-health conditions.

It is still an open question whether to consider the ICF qualifiers "8 – not specified" and "9 – not applicable" missing values represents the best strategy to cope with these response alternatives. The qualifier "8 – not specified" is used when the available information does not suffice to quantify the severity of the problem and "9 – not applicable" when a determine category is not applicable to a patient. These qualifiers are very useful from a clinical point of view but they represent a 'barrier' which is difficult to overcome with parametric statistical methods from a research or statistical point of view. Therefore, alternatives to how the information currently recorded with the qualifiers 8 and 9 could be recorded without using response alternatives that are part of the qualifiers' scale that ranges from 0 to 4 should be considered.

The question whether item one of the SF-36 is the appropriate external standard for the study presented here has to be posed within this context. As mentioned in the introduction, this item has the advantage of being straight, simple and intuitive. However, it also presents different challenges because it is subjective and very broad. One could even argue that, since the concept of health is subjective and represents something different for each different person, one does not know at the end what is assessed with an item like item one of the SF-36. Thus, one does not know at the end what is explained when a regression analysis is performed using such an item. Similar analyses have to be performed with alternative Items and/or indices in future studies.

## Conclusion

A method for selecting candidate ICF categories for a *Generic ICF Core Set*, as well as a number of candidate ICF categories for such a Core Set, is proposed in this study. There are still, however, a number of limitations that have to be overcome and further studied in future investigations. The results of this study also support the opinion that the process of developing a *Generic ICF Core Set *has to be iterative and involve different criteria and methodological approaches. The ultimate aim is to come up with an instrument able to compare disability across health conditions in a uniform way.

## Competing interests

The author(s) declare that they have no competing interests.

## Authors' contributions

AC participated in the conception of the study, was responsible for the training of the persons collecting the data, performed the data analyses and drafted the manuscript. SG was responsible for the data management and monitoring and participated in the data analyses. SC, NK and BTÜ participated in the conception and design of the study. GS conceived the study and advised on the statistical analyses. All the authors reviewed the manuscript before submitting and approved it.

## Pre-publication history

The pre-publication history for this paper can be accessed here:


